# Fast-Response Micro-Phototransistor Based on MoS_2_/Organic Molecule Heterojunction

**DOI:** 10.3390/nano13091491

**Published:** 2023-04-27

**Authors:** Shaista Andleeb, Xiaoyu Wang, Haiyun Dong, Sreeramulu Valligatla, Christian Niclaas Saggau, Libo Ma, Oliver G. Schmidt, Feng Zhu

**Affiliations:** 1 Material Systems for Nanoelectronics, Chemnitz University of Technology, 09107 Chemnitz, Germany; c.n.saggau@ifw-dresden.de (C.N.S.); oliver.schmidt@main.tu-chemnitz.de (O.G.S.); 2 Leibniz-Institute für Festköper- und Werkstoffforschung Dresden, 01069 Dresden, Germany; xiaoyuwangsci@163.com (X.W.); donghaiyun@iccas.ac.cn (H.D.); srihcu08@gmail.com (S.V.); l.ma@ifw-dresden.de (L.M.); 3 Research Center for Materials, Architectures, and Integration of Nanomembranes (MAIN), Chemnitz University of Technology, 09126 Chemnitz, Germany; 4 Department of Physics, School of Science, Hainan University, Haikou 570228, China; 5 School of Science, Dresden University of Technology, 01069 Dresden, Germany; 6 State Key Laboratory of Polymer Physics and Chemistry, Changchun Institute of Applied Chemistry, Chinese Academy of Sciences, Changchun 130022, China

**Keywords:** transition metal dichalcogenides, MoS_2_, organic molecule, VOPc, phototransistor, heterostructure

## Abstract

Over the past years, molybdenum disulfide (MoS_2_) has been the most extensively studied two-dimensional (2D) semiconductormaterial. With unique electrical and optical properties, 2DMoS_2_ is considered to be a promising candidate for future nanoscale electronic and optoelectronic devices. However, charge trapping leads to a persistent photoconductance (PPC), hindering its use for optoelectronic applications. To overcome these drawbacks and improve the optoelectronic performance, organic semiconductors (OSCs) are selected to passivate surface defects, tune the optical characteristics, and modify the doping polarity of 2D MoS_2_. Here, we demonstrate a fast photoresponse in multilayer (ML) MoS_2_ by addressing a heterojunction interface with vanadylphthalocyanine (VOPc) molecules. The MoS_2_/VOPc van der Waals interaction that has been established encourages the PPC effect in MoS_2_ by rapidly segregating photo-generated holes, which move away from the traps of MoS_2_ toward the VOPc molecules. The MoS_2_/VOPc phototransistor exhibits a fast photo response of less than 15 ms for decay and rise, which is enhanced by 3ordersof magnitude in comparison to that of a pristine MoS_2_-based phototransistor (seconds to tens of seconds). This work offers a means to realize high-performance transition metal dichalcogenide (TMD)-based photodetection with a fast response speed.

## 1. Introduction

MoS_2_ is a transition metal dichalcogenide (TMD) semiconductor with excellent optoelectronic properties [1,2,3,4]. With their unique and interesting physical properties, such as thickness-dependent energy band gap, stacking structures, and giant magnetoresistance (GMR), two-dimensional (2D)-layered van der Waals (vdW) materials have received a lot of attention. MoS_2_ is a promising TMD material for low-power devices due to its intrinsic band gap of 1.2–1.9 eV [5,6]. Due to the synthesis of numerous 2D-layered vdW materials, such as TMDs, and the development of innovative electronic/optoelectronic applications, the field of 2D vdW materials has progressed significantly [7,8,9,10].

In terms of photodetectors with PPC, MoS_2_-based devices face many issues, such as trap states in MoS_2_ and minority carrier capturing by absorbents [11]. Although minority carrier trapping can play an essential role in enhancing the gain mechanism with a long carrier lifetime, the device reaction time is drastically reduced since the PPC effect usually lasts for hours [12,13]. However, an optimization of the response dynamics, for example, a modification of the density of states, surface passivation, or field-effect approaches, results in the loss of sensitivity because trap-induced gain processes are adversely suppressed [14,15,16]. The well-established photodetectors with carrier segregation and capturing at the interfaces contribute to optimizing the detection bandwidth and sensitivity [17].

As a result of the rapid photovoltaic charge transfer in the presence of an internal electric field and type II band alignment, fast gain mechanisms are obtained. However, material growth and device integration, with the predominant vdW connections, are still challenging. Nanocrystal devices, for example, frequently suffer from stability problems [18]. For the creation of 2D TMD-based detectors, an innovative acceptable charge-transfer interface construction on the 2D surfaces is extremely appealing [19,20]. The atomically thin nature of 2D TMDs enables charge-transfer coupling with nearby substrates and surface absorbents, such as atmospheric O_2_ and moisture. Due to this feature, it is conceivable to tune the properties of the TMD by exploiting surface-assembled charge transport systems [21,22]. Organic molecules that can interact with the interface of 2D TMDs have been explored intensively to tailor the doping polarity through surface defects [23] and tune the photoluminescence characteristics of 2D TMDs. Fundamental organic photovoltaic materials have been demonstrated to exhibit rapid charge couplings with 2D TMDs [24,25]. The organic molecules with a π-conjugated system enhance the charge transport path, improving the photo response behavior of 2D TMD materials [26,27].

In this work, a platform is proposed to achieve fast photo response dynamics inmultilayer (ML) MoS_2_ phototransistors by the deposition of vanadyl phthalocyanine (VOPc) organic molecules. Vanadyl phthalocyanine (VOPc) possesses the highest third-order nonlinear optical susceptibility and the fastest optical response (less than 10 ps) [28]. Because of its high mobility (1 cm^2^/(Vs)), it has been used in the creation of an organic field effect transistor. As a result, we investigated its potential use as an active material in the development of micro phototransistor heterojunction devices [29]. The deflection of the electrons from the MoS_2_ induced by the assembly of VOPc molecules compensates the intrinsic electron doping effect on the surface of the MoS_2_. This charge transfer mechanism allows the generation of electron-hole pairs under illumination, guiding the holes to the VOPc molecules and suppressing the trapping of minority carriers at the substrate that reduces the potential, leading to an increased reaction rate.

## 2. Experimental

### 2.1. The Characterizations of ML MoS_2_

Using the Scotch tape method, a ML MoS_2_ was mechanically cleaved from a MoS_2_ bulk crystal (SPI Supplies) and transferred onto a silicon substrate. The device with a ML of MoS_2_ was heated to 250 °C for 2 h under aN_2_ gas atmosphere (100 sccm flow) to remove impurities and enhance the interfacial adhesion to the substrate. Photoluminance (PL) and Raman measurements were carried out at ambient temperature. A blue laser $\left(\lambda =458\;\mathrm{nm}\right)$ was used as an excitation source and the signal was collected usinga confocal setup (Lab RAM HR Evolution, Horiba, Kyodo, Japan). The laser beam had a spot size of 0.77μm (10 × 0.25: working distance 10 mm). The laser power was ≤5 kW·cm^−2^ to suppress a thermal degradation of the sample.

### 2.2. Micro-Photo FETs Fabrication and Measurements

MoS_2_ MLs were exfoliated from bulk crystals of molybdenite (SPI Supplies) usingthe Scotchtape method and then transferred onto Si/SiO_2_ (1 μmSiO_2_) substrates. A photoresist (AZ-5214E) was then spin-coated at 4500 rpm for 45 s, leading to a 1 μm thickness, and baked at 90 °C for 5 min. After patterning the chip with standard photolithography, Cr/Au (10/50 nm) film was deposited with a deposition rate of 0.5 nm min^−1^ to develop the contact electrodes using a thermal evaporator system. The micro-photo FETs were characterized in a probe station (Form Factor GmbH EPS150RF, Thiendorf, Germany) employing an oscilloscope (VOLTCRAFT DSO-1254F, Conrad Electronic, Germany) and a function generator (Tektronix AFG 3252, Instrumex GmbH, Sauerlach, Germany) (Figure S2b). All measurements were performed under ambient conditions.

### 2.3. Growth of VOPc Thin Films

In a physical vapor deposition (PVD) system (Moor field Minilab 060, Moorfield Nanotechnology Limited, Knutsford, Cheshire, UK), the organic semiconductor VOPc (Sigma–Aldrich Chemie GmbH, Taufkirchen, Germany) was deposited, while the samples were kept at ambient temperature. The VOPc was deposited at a rate of 0.02–0.03 nm min^−1^ at 10^−5^ Pa. AFM (Bruker Icon Agilent 5500, tapping mode, Athens, Greece) was used to obtain the topographical image of the sample.

## 3. Results

**Figure 1:**
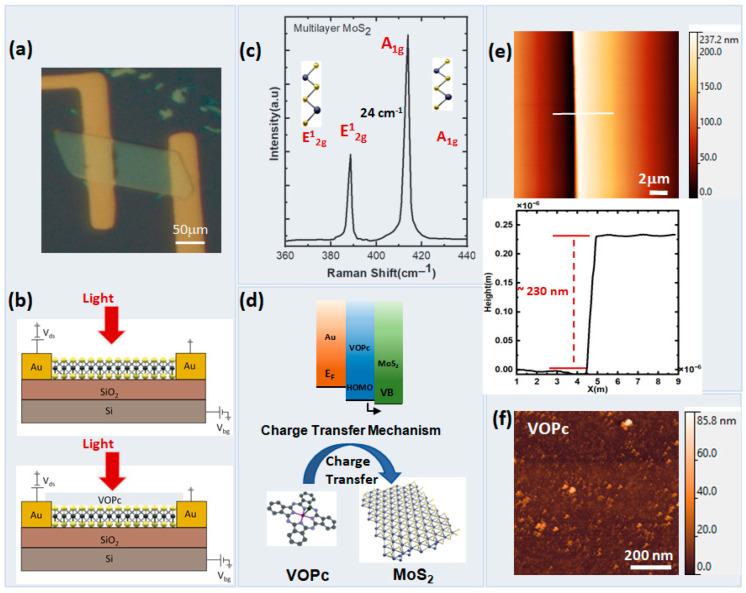
Fabrication of micro-phototransistor. (**a**) Optical image of a fabricated MoS_2_ phototransistor. (**b**) Schematic diagrams of pristine (**top**) and MoS_2_/VOPc (**bottom**) heterojunction phototransistors. (**c**) Raman spectra of the ML MoS_2_ phototransistor. (**d**) Charge transfer mechanism at the interface of MoS_2_ and VOPc. (**e**) AFM image and corresponding cross-section profile of the MoS_2_ device. (**f**) Morphology of VOPc organic molecule.

In this work, to create the vdW molecules2D TMDs heterojunction, the ML MoS_2_ was utilized to investigate the optical properties as a phototransistor. The optical image of the multilayer MoS_2_ phototransistor is shown in Figure 1a. The microstructural fabrication process of a MoS_2_/VOPc heterojunction phototransistor is depicted in Figure S3. Figure 1b shows the devices of a 2D TMDs and a vdW molecule TMD heterojunction phototransistor. The Si substrate acts as a back gate for the field-effect modulation. Evidence of being a multilayer MoS_2_ is supported by the Raman spectroscopy measurements (Figure 1c). The Raman spectra of ML MoS_2_ show two characteristic peaks, named E^1^_2g_ (in-plane vibration) and A_1g_ (out-of-plane vibration), at 389 and 413 cm^–1^, respectively [30]. The difference between these two Raman modes (Δ = A_1g_ − E^1^_2g_) is approximately 24 cm^–1^, reveali ng multilayers [31].

How the charge is transported at the interface of MoS_2_ (n-type semiconductor) and VOPc (p-type organic molecule) is a key phenomenon to enhance the performance and understand the mechanism of a hetero phototransistor device. In 2D TMD heterojunctions containing VOPc, a distinct charge transfer mechanism was observed [32,33]. Due to this effect, electrons are transferred from VOPc to MoS_2_. In the dark state, the electrons and holes recombine at the interface, respectively, driving the energy band bending. In the dark state, the accumulation of electrons and holes at the interface leads to the recombination of these charge carriers, which drives the energy band bending. The energy band bending occurs because the accumulation of charge carriers at the interface changes the local electric field, which affects the distribution of energy levels in the semiconductor layers. VOPc, having the highest occupied molecular orbit (HOMO) level of approximately 5.1 eV, provides an energy path for the holes Furthermore, VOPc organic molecule, with a HOMO level of approximately 5.1 eV, provides an energy path for the holes. This means that the energy levels in VOPc are such that holes can easily move through the material. (Figure 1d) [33,34,35]. The height of MoS_2_ (~230 nm) is measured using atomic force microscopy (AFM) (Figure 1e). The morphology of VOPc molecules (~3 nm) shows the full surface coverage (Figure 1f). The morphology of the film is non-uniform and coarse with particle-like features [36]. Non-uniform and particle-like features indicate the VOPc deposition [37]. The measurements ofthe PL emission and transmittance show the optical characteristics of MoS_2_ (Figure S1) [33,38].

Shifting the binding energy corresponding to defective/sub-stoichiometric 2D materials is an essential factor in understanding the heterojunction effect, which is the primary mechanism directing the charge transfer interface between the organic and 2D materials [32,38]. Forinstance, in the case of the ZnPc-covered MoS_2_ surface, the XPS spectra of pristine MoS_2_ show the main regions (Mo 3d, S 2p). These two characteristic peaks lie at 229.4 and 232.5 eV in the XPS spectrum [33,39]. From pristine MoS_2_ to ZnPc organic-molecule-covered MoS_2_, the binding energy is shifted from 0.22 to 0.25 eV, which indicates the charge transfer phenomenon from the organic molecules to the 2D materials [39].

The type-II band alignment present in most heterostructures with a single layer (SL) MoS_2_ enables the transfer of electrons from the lowest unoccupied molecular orbit (LUMO) of the metallated phthalocyanine (MPc) to the conduction band minima (CBM) of MoS_2_, and of the holes to the highest occupied molecular orbit (HOMO) of the MPcs under photoexcitation. Recently, in the case of VOPc-coupled SL MoS_2_, PL spectra revealed a blue shift for VOPc from 875 nm to 865 nm and a red shift for MoS_2_ from 660 nm to 674 nm [40]. These results indicate a reduction of the bandgap of MoS_2_ due to a lower concentration of free radicals in the depletion region, and a charge transfer from the organic molecules to MoS_2_ [40]. Additionally, negative ground state bleach measurements reveal a specific negative signal in the heterojunction above 730 nm, absent from the original SL MoS_2_ layer. After photoexcitation, the signal originates from the active interface between MoS_2_ and VOPc [40].

Ultraviolet photoelectron spectroscopy is used to reveal the band alignment at the junction, providing additional proof of the heterojunction effect. The Fermi level (E_F_) is used to describe the binding energy in UPS spectra. In the case of ZnPc-covered MoS_2_, it was reported that the valence band maximum (VBM) of pristine and ZnPc-covered MoS_2_ lies at 1.45 and 1.2 eV below E_F_, respectively [35,41]. Shifting of the VBM towards E_F_ in ZnPc-covered MoS_2_ illustrates that electron doping in MoS_2_ is mitigated due to ZnPc molecules [33,42].

**Figure 2:**
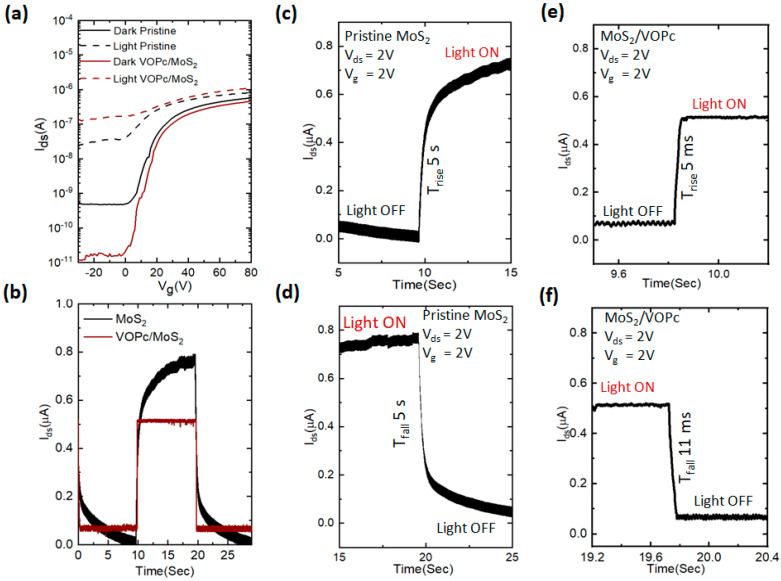
Electric and photo measurements of micro-phototransistor. (**a**) Combined transfer curves of pristine MoS_2_ and MoS_2_/VOPcheterojunction phototransistor. (**b**) Combined responsivity of pristine MoS_2_ and MoS_2_/VOPcheterojunction phototransistor. (**c**,**e**) Photoresponse rise time of pristine MoS_2_ and MoS_2_/VOPcheterojunction phototransistor, respectively. (**d**,**f**) Photoresponse decay time of pristine MoS_2_ and MoS_2_/VOPcheterojunction phototransistor, respectively.

For a better understanding of the charge transfer interaction between VOPc and MoS_2_, the photoresponse behavior of VOPc-covered MoS_2_ detectors is vital. First, the MoS_2_ device’s field-effect transfer curves are measured before and after depositing the VOPc molecules (Figure 2a). With a source-drain bias of (V_ds_) and a gate voltage (V_g_) of 2 V, the measurements were performed in the dark and under illumination (530 nm, 5 mW/cm^2^). The measured transfer curves show the n-type conduction due to the Fermi level pinning effect, demonstrating the electrons as majority charge [41,42,43]. The MoS_2_ phototransistor with VOPc shows a reduction of the source-drain current (I_ds_) and the threshold voltage (V_th_) shifts toward the positive direction. Furthermore, a pronounced electron compensation effect is observed under dark and illumination conditions. The n-type conduction in the positive direction demonstrates that the main cause of the trapping of the photo-generated holes in comparison to electrons in MoS_2_ is a significant n-type photo-doping effect [44,45].

A direct comparison of the response dynamics for the pristine MoS_2_ and ~3 nm VOPc-coveredMoS_2_ is shown in Figure 2b. The photoresponse persists for seconds for the pristine MoS_2_ (Figure 2b). This phenomenon was already studied in exfoliated (mechanically cleavage) and direct-grown (CVD) MoS_2_, where it was suggested that the slow hole capturing and releasing originates due to defect states or as a consequence of substrate interactions [42,46]. By contrast, the devices with VOPc molecules coupled to the surface of MoS_2_ show a photo response improvement. A steep rise and decay of the photoresponse is observed under on and offlight conditions as seen in Figure 2e,f. The mobilities, the charge carries’ densities, and the threshold voltages of pristine MoS_2_ and MoS_2_/VOPc heterojunction phototransistors lie between 47 cm^2^/Vs and 27 cm^2^/Vs, 2.7 × 10^10^ cm^2^ and 0.6 × 10^10^ cm^2^, and 19 V and −5 V, respectively, in the dark and under illuminated conditions (Figure S4) [47].

Figure 2b illustrates how adding VOPc molecules to the surface increases the photo response kinetics in the phototransistor. After the device is covered by VOPc, it exhibits a sharp photocurrent rise and decline in the dark and under illumination. Figure 2c–f compares the response dynamics for pristine MoS_2_ with that of VOPc-covered MoS_2_ (~3 nm). The photo response of pristine MoS_2_ is seen to last for many seconds. The effect is comparable to PCC, previously observed in both mechanically and CVD-grown MoS_2_. In both cases, the effect was related to the slow minority hole entrapment and their decay at the contested surface-absorbed molecules, defect states, and substrate interfaces. In both cases, the PCC effect has been related to the slow minority hole entrapment and their decay at the interface between the MoS_2_ layer and the substrate, as well as at surface-absorbed molecules and defect states. [48,49]. Here, we show that the slow photo response dynamics of MoS_2_ can be considerably improved by covering it with~3 nm VOPc, leading to the fast rise and decay times of 5 and 11 ms, respectively. Figure 2e,f enhanced the photoresponse by three orders of magnitude compared to that of pristine MoS_2_ [50].

**Figure 3:**
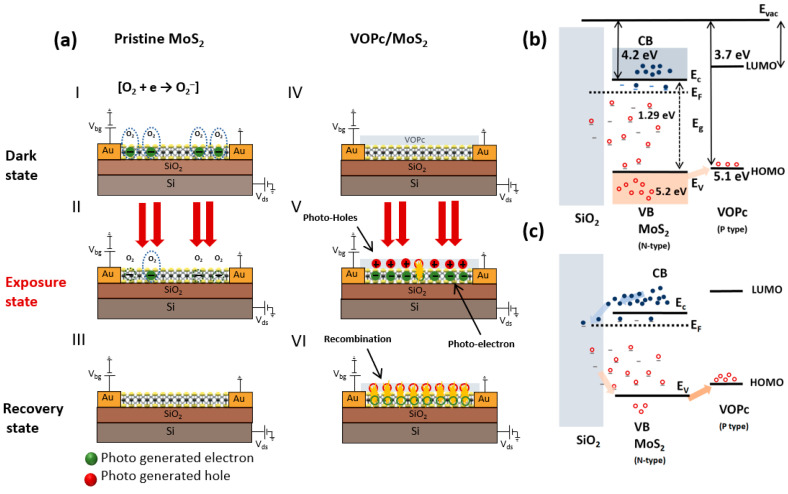
Photoresponse phenomenon and energy band diagram of micro-phototransistor. (**a**) Phenomenon of the light at the interface of pristine MoS_2_ (panels (I, II, III)) and VOPc/MoS_2_ (panels (IV, V, VI)) heterojunction phototransistor. Panels (I, IV) in dark state, panels (II, V) in exposure state, and panels (III, VI) in recovery state. (**b**) Energy band diagram of Si substrate, MoS_2_, and VOPc molecules. (**c**) Generation of holes by applying the positive gate bias in VOPc/MoS_2_ heterojunction phototransistor.

The photo-generated carrier relaxation processes in pristine and heterojunction phototransistors are illustrated in Figure 3a. In the pristine MoS_2_ FETs, oxygen molecules attach to the MoS_2_ surface electrons $\left[O_{2}+e\to O_{2}^{-}\right]$ with a weak binding energy, leading to a notable decrease in the MoS_2_ channel conductivity (panel I) [51]. However, under laser irradiation, the absorbed oxygen molecules can desorb, reducing the trap states, and resulting in an enhancement of the conductivity of the active MoS_2_ region (panel II) [51]. This persists until the oxygen molecules reabsorb. The photo-generated electrons predominantly contribute to an increased current in the pristine MoS_2_ phototransistor, as opposed to the photo-generated holes, which move along the active region at the surface for the interaction with the melectrons (panel III in Figure 3a) [51]. which show the gradual enhancement of the drain current to the saturation area in the laser pulse irradiation This process results in a gradual enhancement of the drain current to the saturation area in the laser pulse irradiation.. An accumulation of the photo-generated electrons continues in the active areauntil anequilibrium stateis achieved after multiple absorption and desorption cycles of oxygen molecules. After a number absorption and desorption cycles of oxygen molecules, photo-generated electrons continue to accumulate in the active area until an equilibrium state is achieved [52,53].

By contrast, in the VOPc-covered MoS_2_ FET, the oxygen molecules are unable to interact with the surface. Due to the disparity in the carrier concentrations, the main carriers of each layer (i.e., holes in p-type VOPc and electrons in n-type MoS_2_) recombine at the contact surface in the absence of light (panelIV), hence, forming a depleted region and resulting in a reduced dark current (as shown in Figure 2a). Due to the absence of oxygen molecules in the active area during illumination, the drain current can attain the saturation mode and ramp up quickly (see Figure 2b). During the laser irradiation, an equal number of photo-generated electrons and holes constantly accumulate at the contact surface of MoS_2_ and VOPc (panel V). As the light is switched off, the photo-generatedholes in VOPc film and electrons in MoS_2_ quickly recombine (panel VI) [40].

Figure 3c shows schematic band diagrams of MoS_2_ and VOPc having the reported energy levels of VB, CB, HOMO, and LUMO of 5.2 eV, 4.2 eV, 5.1 eV, and 3.7 eV, respectively [40]. The charge transfer from VOPc to MoS_2_ leads to different Fermi levels. In the proximity of the MoS_2_, the confined holes of the organic molecules accumulate, creating an internal electric field at the interfaces [40,49], promoting interfacial band bending and allowing the electrons to tunnel from MoS_2_ to VOPc molecules, resulting in a photo-excited exciton separation. Due to the device’s charge neutrality, the direct photo-generated electron-hole pairs and free electrons make up the equilibrium photocurrent, which is the counterbalance of the holes.

As shown in Figure 3c, after a forward bias voltage is applied, the electrons accumulate in the MoS_2_ region and at the interface with the SiO_2_ substrate, while the holes are pushed out. Following the breakdown of the free electron and hole equilibrium by an external gate bias, the holes begin to escape from the trap states until a new equilibrium is achieved. Thus, by measuring the transient current, it is possible to determine the escape time constants for the holes under an illumination.

**Figure 4:**
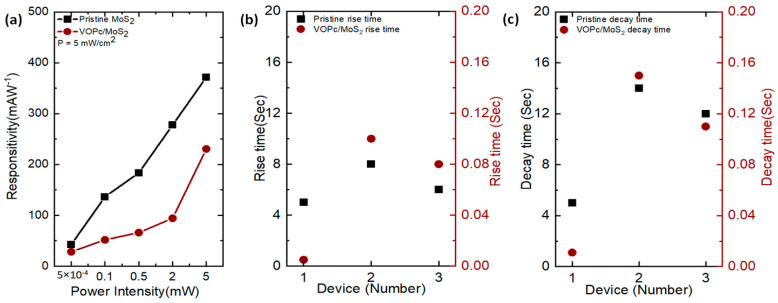
Photo responsivity and response time of micro-phototransistor. (**a**) Power intensity-dependent responsivity of pristine MoS_2_ and MoS_2_/VOPcheterojunction phototransistor. (**b**) The photo rise time of three pristine MoS_2_ and VOPc/MoS_2_ heterojunction phototransistor devices. (**c**) The photo decay time of three pristine MoS_2_ and VOPc/MoS_2_ heterojunction phototransistor devices.

Figure 4a depicts the power intensity-dependent responsivity of the phototransistor device’s performance. The responsivity of a phototransistor device can be expressed by

(1) $$R=\frac{I\;illum-I\;dark\;}{P\;illum}$$

where Iillum, Idark, and Pillum are illuminated, dark current, and power of the light that illuminates the operating region of the device [34].

The responsivity was calculated at different power intensity values. For the pristine and VOPc-covered MoS_2_ at the lowest light intensity (500 nW/cm^2^), the responsivity (R) is 380 and 240 mA/W, respectively. It is seen that the R steadily increases for both devices as the light intensity increases. The photocurrent vs. power intensity can be fitted to a power law dependence of the form I_ph_α P^1.1^ (Figure S2a). Within comparison to other 2D material hetero and hybrid structures, we fabricated fast ML phototransistors (Table S1) [35,51,54,55,56].

To determine the reproducibility and an average rise and decay duration, three micro-phototransistors were measured in Figure 4b,c. The statistics of the reaction time from 20 MoS_2_ phototransistors are shown in Figure S5.

The response time range is less than 200 ms, as shown in Figure 4b,c. The uncontrollable device fabrication process generates volatility in the MoS_2_ phototransistor’s reaction time. The different channel areas, thickness deviations, contamination of the device surface, and device contact resistance cause several phenomena that impair the performance of the devices. To perform consistently, the MoS_2_ phototransistors need to improve the contacts by reducing the contact resistance to create a smooth uniform channel area and thickness. To improve the performance of MoS_2_ phototransistors, it is necessary to reduce the contact resistance and create a smooth and uniform channel area and thickness. However, the development of the heterostructure formed by 2D materials and organic molecules is highly challenging, given these limitations. To overcome these challenges, we developed hetero junction phototransistors exhibiting a fast response time. This significant improvement demonstrates that overcoming the limitations that have been highlighted will significantly enhance the performance of the unique 2D materials and organic phototransistors.

## 4. Conclusions

In conclusion, we showed a heterojunction device as a planar phototransistor with a quick response mode that can be used in several ways. By efficiently constructing van der Waals interfaces with organic VOPc molecules, the MoS_2_ phototransistor exhibited a three-order of magnitude improvement of the photo response dynamics compared to the reported work in Table S1 [35,51,54,55,56]. Our 2D/organic heterojunction phototransistor will enable the production of high-performance versatile devices. As a result, the method has the potential to produce detectors with quick response times needed for practical applications. The work proves the successful application of TMD/organic heterojunction materials for improvements in the optoelectronic capabilities of 2D TMDs for photodetection.

## Data Availability

The data presented in this study are available upon request from the corresponding authors.

## Supplementary Materials

The following supporting information can be downloaded at: https://www.mdpi.com/article/10.3390/nano13091491/s1, Figure S1: (a) Absorption spectra of ML MoS2. (b) Transmittance spectra of ML MoS2.; Figure S2: (a) Power intensity-dependent photocurrent measurements. (b) Measurement Setup of the phototransistor; Figure S3: Schematic diagram of the device fabrication process and optical images (a) Schematic diagram of MoS2 device fabrication process with all steps, see Figure S3. (b) Optical image of device Cr/Au electrodes, see Figure S3. (c) Optical image of MoS2 with Cr/Au electrodes, see Figure S3. (d) Optical image of MoS2 device; Figure S4: (a) Carrier densities of Pristine (P) MoS2 and VOPc/MoS2 heterojunction (H) phototransistor devices in dark and light conditions. (b) Mobilities and the threshold voltages [47] of the Pristine (P) MoS2 and the VOPc/MoS2 heterojunction (H) phototransistor devices in dark and light conditions; Figure S5: Statistics data of phototransistors (a) Statistics data of photo rise time from 20 MoS2 phototransistors. (b) Statistics data of photo decay time from 20 MoS2 phototransistors; Table S1: State of Art of the response time of VOPc/MoS2 heterojunction phototransistor. Refs. [35,43,51,54,55,56] are cited in supplementary materials.
