# Supplementary material for: Fast-Response Micro-Phototransistor Based on MoS2/Organic Molecule Heterojunction
Source: Nanomaterials (Basel). 2023 Apr 27;13(9):1491. doi: 10.3390/nano13091491 (PMC10180112; doi:10.3390/nano13091491)
Supplement: Supplementary file 1 [file nanomaterials-13-01491-s001.zip › nanomaterials-2327447-supplementary.pdf]

Supplementary Material:

Fast-Response Micro-Phototransistor Based on MoS_2/ Organic Molecule Heterojunction

Shaista Andleeb [1,2,3,*], Xiaoyu Wang [2,4], Haiyun Dong [2], Sreeramulu Valligatla [2], Christian Niclaas Saggau [1,2,3], Libo Ma [2], Oliver G. Schmidt [1,2,3,5] and Feng Zhu [6,*]

[1] Material Systems for Nanoelectronics, Chemnitz University of Technology, 09107 Chemnitz, Germany; c.n.saggau@ifw-dresden.de (C.N.S.); oliver.schmidt@main.tu-chemnitz.de (O.G.S.)
[2] Leibniz-Institute für Festköper- und Werkstoffforschung Dresden, 01069 Dresden, Germany;xiaoyuwangsci@163.com (X.W.);donghaiyun@iccas.ac.cn (H.D.);srihcu08@gmail.com (S.V.); l.ma@ifw-dresden.de (L.M.)
[3] Research Center for Materials, Architectures, and Integration of Nanomembranes (MAIN), Chemnitz University of Technology, 09126 Chemnitz, Germany
[4] Department of Physics, School of Science, Hainan University, Haikou 570228, China
[5] School of Science, Dresden University of Technology, 01069 Dresden, Germany
[6] State Key Laboratory of Polymer Physics and Chemistry, Changchun Institute of Applied Chemistry, Chinese Academy of Sciences, Changchun130022, China
* Correspondence: s.andleeb@ifw-dresden.de (S.A.); zhufeng@ciac.ac.cn (F.Z.)

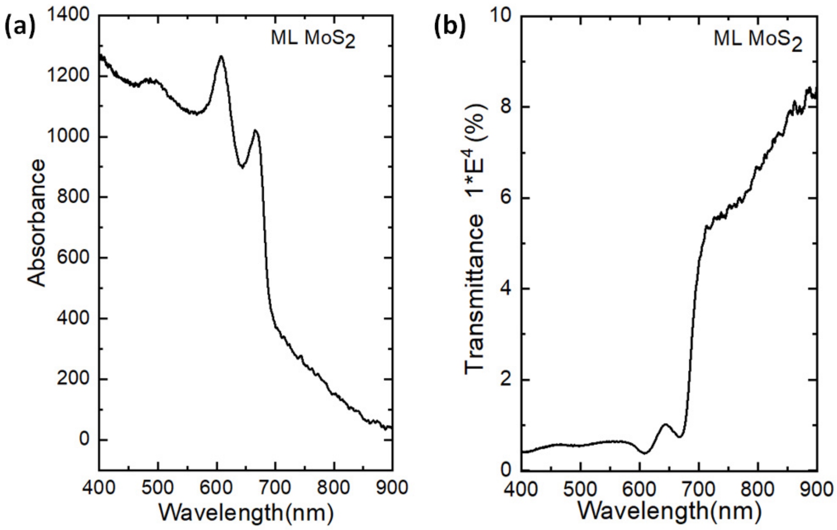

Figure S1. (a) Absorption spectra of ML MoS_2. (b) Transmittance spectra of ML MoS_2.

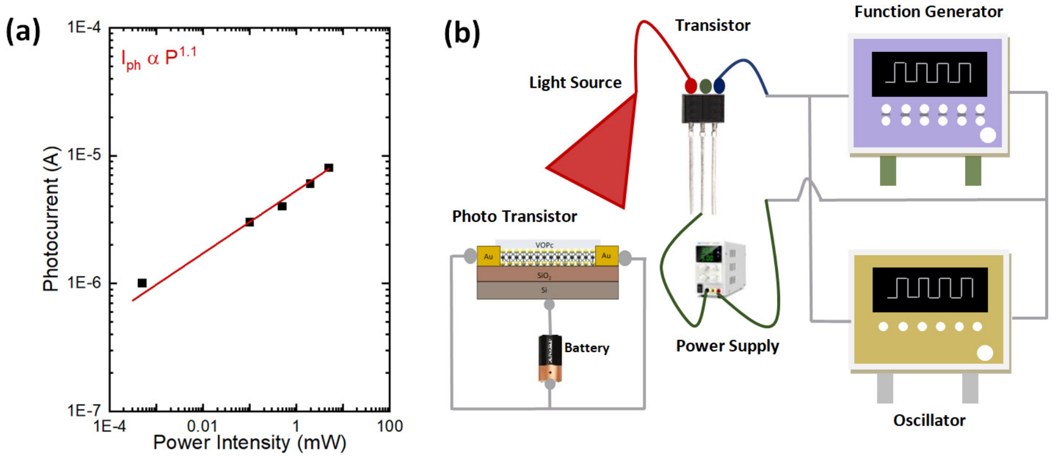

Figure S2. (a) Power intensity-dependent photocurrent measurements. (b) Measurement Setup of the phototransistor.

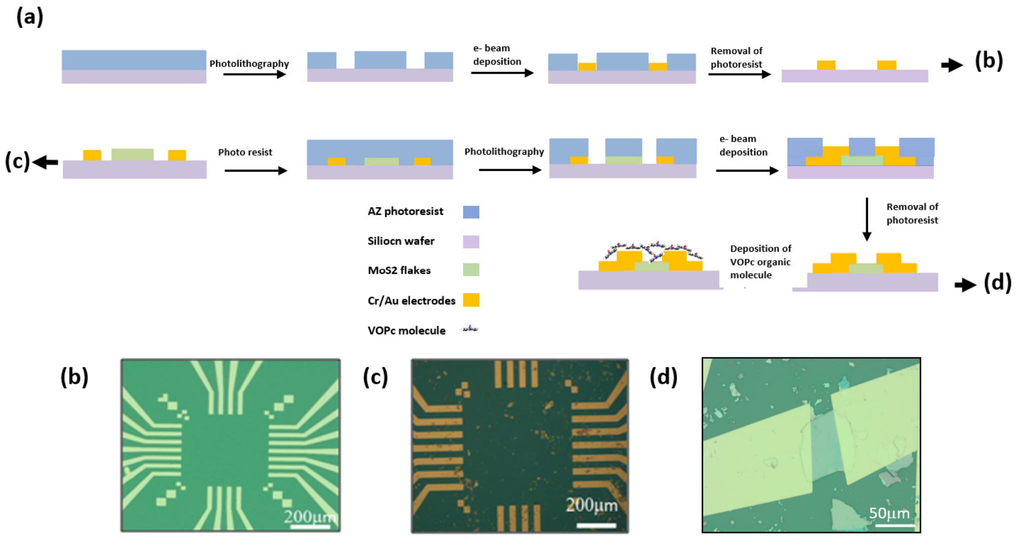

Figure S3. Schematic diagram of the device fabrication process and optical images (a) Schematic diagram of MoS_2 device fabrication process with all steps, see Fig. S3. (b) Optical image of device Cr/Au electrodes, see Fig. S3. (c) Optical image of MoS_2 with Cr/Au electrodes, see Fig. S3. (d) Optical image of MoS_2 device.

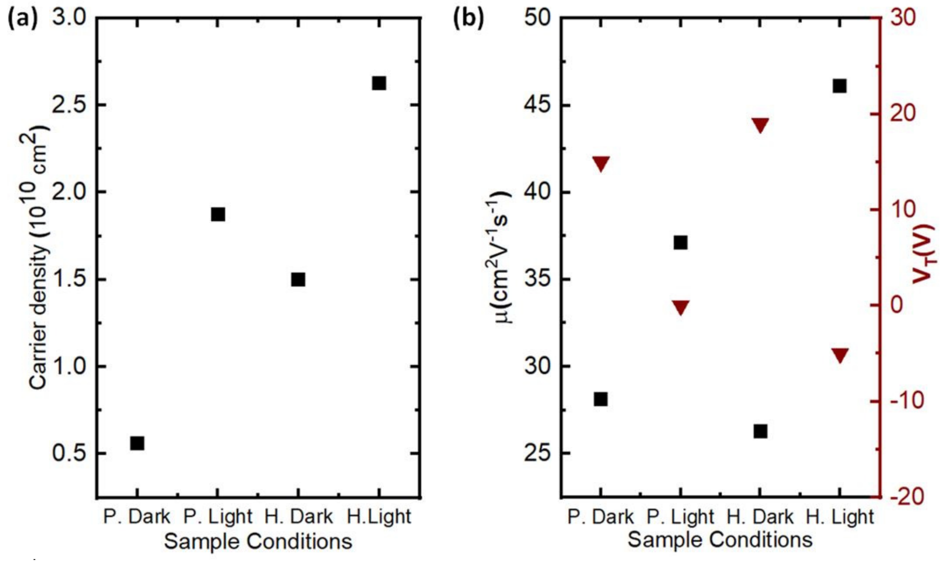

Figure S4. (a) Carrier densities of Pristine (P) MoS_2 and VOPc/MoS_2 heterojunction (H) phototransistor devices in dark and light conditions. (b) Mobilities and the threshold voltages [47] of the Pristine (P) MoS_2 and the VOPc/MoS_2 heterojunction (H) phototransistor devices in dark and light conditions.

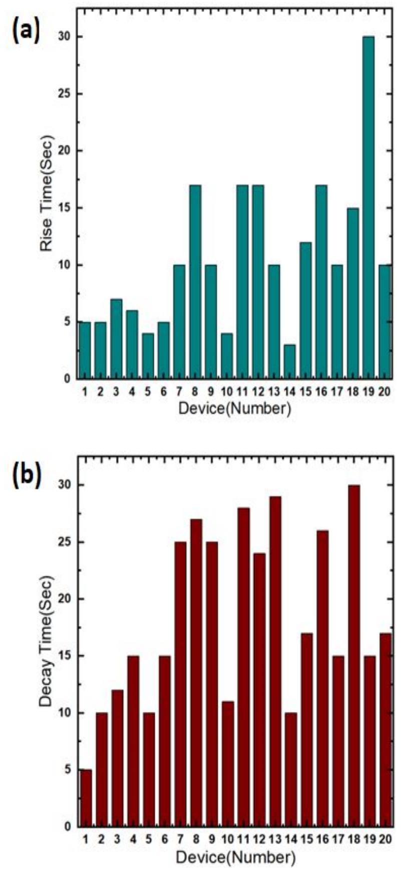

Figure S5. Statistics data of phototransistors (a) Statistics data of photo rise time from 20 MoS_2 phototransistors. (b) Statistics data of photo decay time from 20 MoS_2 phototransistors.

Table S1. State of Art of the response time of VOPc/MoS_2 heterojunction phototransistor.

**Photodetectors Structure**	**Thickness (nm)**	**Photoresponse (Sec)**	**Reference**
CuPc/MoS_2	2	5	[51]
Perovskite/Graphene	SL	5.3	[54]
C8-BTBT/Graphene	SL	0.025	[55]
Perovskite/Graphene	SL	0.54	[56]
ZnPc/MoS_2	SL	0.008	[35]
**Our work**	**230**	**0.011**	**This work**
